# Pulmonary Hypertension in Sickle Cell Disease: A Case Series

**DOI:** 10.7759/cureus.89012

**Published:** 2025-07-29

**Authors:** Ngowari Pokima, Sunydip Gill, Ramtin Moradi, Aye M Thida, John Muthu

**Affiliations:** 1 Internal Medicine, Staten Island University Hospital, Northwell Health, Staten Isand, USA; 2 Internal Medicine, State University of New York Downstate Health Sciences University, Brooklyn, USA; 3 Hematology and Oncology, State University of New York Downstate Health Sciences University, Brooklyn, USA; 4 Department of Medicine, Sickle Cell Division, Kings County Hospital Center, Brooklyn, USA

**Keywords:** complications of sickle cell disease, multifactorial pulmonary hypertension, pulmonary hypertension, sickle cell hbsc, sickle cell trait-associated lung disease

## Abstract

Pulmonary hypertension (PH) is a serious and increasingly recognized complication in sickle cell disease (SCD), particularly in individuals with the homozygous genotype. Elevated tricuspid regurgitant velocity (TRV) increases the potential for future development of PH, yet no standardized management approach currently exists, likely because of the varied pathophysiology of PH in these patients. Transthoracic echocardiogram-derived elevated TRV of more than 2.7 m/second in patients with SCD is associated with increased mortality and remains the only non-invasive approach to diagnosis. PH in patients with SCD can be due to various etiologies and can be considered either precapillary or post-capillary PH. This case series highlights the need to diagnose the underlying etiology of SCD-induced PH, which in turn will dictate appropriate management. Additionally, this case series will review recent management strategies available for treating patients with established PH and address significant risk factors for patients at risk for the development of PH.

## Introduction

Pulmonary hypertension (PH) is an increasingly recognized complication of sickle cell disease (SCD), associated with significant morbidity and mortality. An elevated tricuspid regurgitant velocity (TRV) ≥ 2.5 m/s, elevated N-terminal pro b-type natriuretic peptide (NT-proBNP) levels (≥ 160 pg/mL), or confirmed PH via right heart catheterization (RHC) (mean pulmonary arterial pressure (mPAP) > 20 mmHg) identifies high-risk patients [1]. The pathophysiology of PH in SCD is complex, involving hemolysis, endothelial dysfunction, thromboembolism, and left heart disease, leading to varied PH classifications (Groups 1-5) [2]. No standardized management approach exists due to this heterogeneity. This case series illustrates the diverse etiologies of SCD-associated PH, highlights diagnostic challenges, and reviews management strategies to improve outcomes in this high-risk population.

## Case presentation

Case 1

A 64-year-old woman with homozygous SCD (HbSS and 5% baseline fetal hemoglobin) was diagnosed with PH. Her PH was considered to be multifactorial as, in addition to SCD, she had a combination of left-sided valvular heart disease (mild to moderate aortic and mild mitral valve incompetence with an ejection fraction (EF) of 60% and left atrial dilation) and a history of pulmonary embolism diagnosed years before presentation. The PH as a result of left heart disease is categorized as a Group 2 PH, and her PH as a result of pulmonary embolism is categorized as a Group 4 PH [3-5]. Her most recent echocardiogram in 2023 revealed moderate PH with a calculated mean pulmonary artery pressure (mPAP) of 39 mmHg. The patient continued treatment for pulmonary embolus with oral anticoagulants in addition to standard hydroxyurea therapy at her maximal tolerated dose of 500 mg/day, but did not undergo any corrective valve surgery for her left-sided valvular heart disease. She received no additional treatments for her sickle cell disease, nor was she on any other medications. At the subsequent follow-ups, she showed symptomatic improvement in her PH with the resolution of hypoxemia and a decrease in mPAP to 18 mmHg. No additional SCD therapies or medications were prescribed. This case demonstrates that optimizing SCD management with hydroxyurea can improve PH, likely by reducing hemolysis-driven endothelial dysfunction, even without addressing mild valvular disease.

Case 2

A 25-year-old woman with homozygous sickle cell disease (HbSS and 22% baseline fetal hemoglobin) presented with dyspnea and was found to have severe mitral stenosis and moderate aortic stenosis, likely rheumatic in etiology, with mild PH; her mPAP was measured to be 26 mm Hg. Additionally, her echocardiogram showed an EF of 55% and left atrial dilation. This PH is categorized as a Group 2 PH [3-5]. She underwent mitral and aortic valve replacements presented with normalization of PH with mPAP of 20 mmHg. She is maintained on lifelong warfarin for prosthetic metallic mitral and aortic valves and a maximal tolerated dose of hydroxyurea (1,500 mg three times weekly and 2,000 mg on alternating days). This patient did have a history of non-adherence to her medications, with the patient experiencing vaso-occlusive crises (VOCs) during periods of non-adherence. Post surgery, there was a significant improvement in her dyspnea. No additional SCD therapies or medications were prescribed. This case highlights the critical role of managing structural heart disease while treating PH, with hydroxyurea supporting SCD management by reducing VOCs and hemolysis.

Case 3

A 28-year-old woman with homozygous sickle cell disease (HbSS and 7.5% baseline HbF) presented with a BMI of 37 kg/m^2 ^and obstructive sleep apnea, and an mPAP of 28 mmHg. Additionally, her echocardiogram showed an EF of 60% and no left atrial dilation. Her symptoms and mPAP remain steady, and her PH has not progressed with concurrent treatment for SCD with hydroxyurea (2,000 mg/day). This is likely due to the non-addressal of her obstructive sleep apnea (OSA), which we consider to be causative of her PH, specifically a Group 3 PH [3-5]. She received no additional treatments for SCD, nor was she on any other medications. The patient was referred to the pulmonary team to address her OSA, which led to partial symptom improvement, including reduced dyspnea. This case underscores the need for multidisciplinary management, as untreated OSA limited PH improvement despite SCD therapy.

## Discussion

Pulmonary hypertension in SCD

Recent updates in the classification of PH now categorize PH associated with SCD and other chronic hemolytic anemias within Group 5, based on distinct pathological and hemodynamic features observed in these patients [2]. This classification also reflects the uncertainty regarding the efficacy of PH-specific therapies in this population. Research shows that intravascular hemolysis in SCD results in the release of cell-free hemoglobin, which scavenges nitric oxide and disrupts vascular endothelial function [6]. This process is one of the primary contributors to pre-capillary PH in these patients. 

In addition to hemolysis, chronic anemia-induced left ventricular diastolic dysfunction in SCD patients can lead to post-capillary PH, which is categorized under Group 2 PH [5]. This is particularly relevant in patients with left ventricular dysfunction, as seen in Case 1. Furthermore, the oxidative damage caused by hemolysis activates platelets and triggers hypercoagulability, increasing the risk of thromboembolic events such as deep vein thrombosis and pulmonary embolism, which can result in Group 4 PH [5].

The frequency of SCD-induced PH in adults has been reported to range between 6-10% when diagnosed using RHC [6,7]. SCD-induced PH is strongly linked with increased mortality, as highlighted by the high-risk group identified in various studies. These patients, who show elevated TRV ≥ 2.5 m/second, elevated NT-proBNP levels ≥ 160 pg/mL, or confirmed PH based on RHC findings (mPAP > 20 mmHg), face significant risks. Even mild elevations in pulmonary vascular resistance (PVR) are linked with increased mortality [1].

When pulmonary capillary wedge pressure (PCWP) exceeds 15 mmHg, as seen in Cases 1 and 3, it suggests post-capillary PH secondary to left ventricular dysfunction. Since RHC was not done, PCWP was estimated using the Nagueh formula, where PCWP = 1.24 x (E/e') + 1.9, and the E/e' ratio was approximated to be the left atrial pressure as measured on echocardiogram. Management in these situations should focus on addressing left heart pathology and improving overall cardiovascular function. Symptomatic PH in SCD patients frequently presents as dyspnea, often indistinguishable from anemia, making it critical to maintain a high index of suspicion in these patients [8].

Hemodynamic insights into SCD-associated pulmonary hypertension

A review of hemodynamic patterns in SCD-related PH by Gladwin et al. revealed the low positive predictive value of using echocardiography alone for diagnosing PH. While 27% of patients with SCD exhibited TRV ≥ 2.5 m/second, only 6% were confirmed to have PH upon RHC. This highlights the need for RHC as the definitive diagnostic tool for PH, especially for patients with borderline TRV values (2.5 to 2.9 m/seconds) [9]. NT-proBNP levels and the six-minute walk distance were also found to be crucial markers in assessing the severity and prognosis of SCD-induced PH [10]. This further supports integrating these functional markers into screening and management protocols. None of the three patients in the current report underwent catheterization, and although the Nagueh formula was used to approximate the PCWP, RHC is the ideal choice to definitively diagnose PH.

Management of SCD-associated pulmonary hypertension

Given the increased mortality associated with SCD-induced PH, an effective treatment strategy for the different etiologies is crucial. All patients should receive optimized treatment for their SCD to reduce RBC hemolysis and vaso-occlusion. Hydroxyurea, an inducer of fetal hemoglobin (HbF), has been shown to prevent vaso-occlusive episodes, including acute chest syndrome (ACS), which can elevate pulmonary artery pressures [11]. The Multicenter Study of Hydroxyurea in Sickle Cell Anemia (MSH) trial demonstrated that patients receiving hydroxyurea had a decreased incidence of ACS, lower rates of VOC, and longer periods until subsequent crises compared to placebo [12]. All three cases presented in the current report received hydroxyurea as part of their treatment regimen. Case 1’s mPAP reduction (39 mmHg to 18 mmHg) with hydroxyurea alone suggests hemolysis reduction may suffice in multifactorial PH with mild structural disease, although it is likely that treatment of the pulmonary embolism with anticoagulation also contributed to the mPAP decrease. Case 2, which was of the youngest patient with a demonstrated period of non-adherence to hydroxyurea, had more VOC events during periods when she was not taking hydroxyurea per chart review. In Case 2, PH resolution post valve replacement mirrors findings that correcting left heart pathology is critical for Group 2 PH, with hydroxyurea enhancing outcomes by reducing VOCs, unlike non-adherent periods correlating with higher VOCs. In Case 3, stable PH, despite hydroxyurea, underscores the critical need for multidisciplinary care to address both SCD and contributing comorbidities.

Chronic anemia-induced left ventricular dysfunction predisposes all patients with significant anemia to post-capillary PH (Type 2), requiring careful management of both SCD and consequent left ventricular hypertrophy and functional mitral regurgitation. Simultaneously, hemolysis-driven endothelial dysfunction predisposes patients to pre-capillary PH (Type 1) [5]. Managing these patients requires addressing both the hematologic and cardiovascular components of the disease, ensuring that therapies aimed at correcting SCD and controlling pulmonary pressures are balanced and effective.

For patients with RHC findings consistent with precapillary PH, pulmonary arterial hypertension (PAH)-specific therapies, including prostacyclin agonists, endothelin receptor antagonists, and phosphodiesterase inhibitors, may be considered. However, no large-scale, placebo-controlled trials have evaluated the efficacy of these therapies in SCD patients, and their safety remains uncertain. Notably, two clinical trials, ASSET-1 and -2, investigating bosentan, a dual endothelin receptor antagonist, were halted due to sponsorship withdrawal, while the walk-PHaSSt trial assessing sildenafil was terminated early due to an increased incidence of pain crises [13,14].

The role of transfusion therapy in SCD-PH remains controversial. While it may provide benefits in managing SCD complications like stroke and ACS, there is insufficient conclusive data to support its widespread use. Additionally, transfusion therapy complications such as alloimmunization and iron overload contribute to variability in clinical practice. The American Thoracic Society (ATS) recommends chronic red cell exchange (RCE) in patients with high mortality risk who are refractory to hydroxyurea [15]. This recommendation stems from evidence demonstrating the reduction in stroke rates seen in chronic transfusion trials such as the Stroke Prevention Trial in Sickle Cell Anemia (STOP) 1 and 2 [16].

Several studies have also suggested that automated RCE may improve clinical symptoms, hemodynamics, and exercise capacity in patients with pre-capillary PH. A single-institution case series demonstrated that patients undergoing chronic RCE experienced significant improvements in echocardiographic parameters and clinical symptoms, with a relapse of symptoms in one patient who discontinued RCE [17,18].

The ongoing Sickle Cell Disease and Cardiovascular Risk RCE (SCD-CARRE) trial is evaluating the efficacy and safety of chronic transfusion therapy in this population. The results of this trial, expected in December 2025, will likely inform future management guidelines for SCD-induced PH patients at high risk for mortality [19], such as the three cases presented in this report.

## Conclusions

Effective management of PH in SCD requires a multi-pronged approach involving optimized SCD treatment, early diagnosis of at-risk patients, and tailored therapeutic interventions based on other comorbid factors contributing to PH. The ongoing SCD-CARRE trial and the exploration of new pharmacologic agents may shape the future of PH management in SCD. Additionally, further research is necessary to establish the role of subspecialty care and standardized screening in improving outcomes for patients with SCD-induced PH. Continued focus on personalized medicine, addressing comorbidities, and developing novel therapies will be key to advancing care for this high-risk population.
